# Precarious employment and gender-based violence against migrant women: A scoping review mapping the intersections

**DOI:** 10.1371/journal.pone.0337690

**Published:** 2025-12-01

**Authors:** Cyndirela Chadambuka, Prossy Kiddu Namyalo, Rhea Raghunauth, Navya Arora, Fiona Kouyoumdjian, Beverley M. Essue

**Affiliations:** 1 Institute of Health Policy Management and Evaluation, Dalla Lana School of Public Health, University of Toronto,; 2 Faculty of Arts and Science, University of Toronto,; 3 Department of Epidemiology, Mailman School of Public Health, Columbia University,; 4 Department of Family Medicine, McMaster University; National Kaohsiung University of Science and Technology, TAIWAN

## Abstract

The risk of gender-based violence (GBV) against migrant women is largely exacerbated by precarious employment opportunities available to them as they go through the resettlement process. Despite the risk that the connection of precarious employment and GBV pose to migrant women’s health and wellbeing, critical gaps exist in literature. Our scoping review sought to identify and synthesize evidence on the interconnectedness of GBV and precarious employment among migrant women. Six electronic databases were searched for empirical literature and two reviewers independently conducted title/abstract and full text screening of studies that met the inclusion criteria. Data synthesis was guided by the intersectionality theory and the Feminist Political Economy framework. 50 articles met the criteria for inclusion in this review. Our findings reveal that precarious employment plays both a catalytic and consequential role in GBV. Findings highlighted how post-migration shifts in gender roles, schedule unpredictability leading to work-life imbalance, and debt bondage trap migrant women in cycles of exploitation and abuse. Few studies highlighted how human trafficking is intertwined with precarious labor markets, where the exploitation and abuse of migrant women mirror the characteristics of human trafficking. This review underscores the urgent need for integrated policy responses that are not only focused on individual supports but also address the structural drivers or labor precarity and protect migrant women from GBV and human trafficking. By applying an intersectional lens, policies and intervention programs can tackle systemic oppression across economic, and social systems essential in reducing exploitation and abuse to advance migrant women’s wellbeing.

## Introduction

Gender based violence (GBV) remains a significant public health problem. Although prevalent across all genders, women are more likely to experience GBV and bear a disproportionate impact [1,2]. One in three women is reported to experience GBV in her lifetime [2]. Evidence has shown that migrant women are particularly vulnerable to GBV, and their experiences are complex and multi-faceted [3]. This vulnerability to GBV has been linked to precarious employment, which is the type of employment most often available to migrant women, especially in the short term during the settlement period in a new country, and can amplify the susceptibility to abuse and exploitation [4–7]. Precarious employment is characterized by dimensions of employment insecurity, income inadequacy, lack of rights and protection, and workers’ limited autonomy over wages, working hours, and conditions [6,8–11]. Neoliberal policies, characterized by privatization and the reduction of state intervention, have led to significant transformations in the labor market, and these changes have been marked by an increase in the participation of women in the workforce. The emphasis on labor market flexibility and privatization shape gendered vulnerability by pushing migrant women into precarious and informal jobs. For example, International Labour Organization (ILO) estimates that nearly 60% of women work in the informal economy as compared to men (54%) [12] and this concentration in informal jobs exacerbates their risk to exploitation and GBV. Neoliberal policies and reforms have ultimately led to a higher demand for cheap labor and the growth of informal and part-time sectors, where women, including migrant women, are disproportionately represented in precarious jobs that typically offer no protection, less security, lower wages, and few to no benefits [13]. Research has also shown that migrant women experience more unfavorable labor market outcomes, have limited access to credit and education, and encounter more significant gender wage gaps, and thus are disproportionately represented in precarious employment [14–16].

The interconnectedness of precarious employment and GBV among migrant women is evident, as precarious employment can elevate the risk and intensity of GBV, while GBV can push women into precarious employment. Studies conducted have shown how various factors, such as poverty, can force women into accepting low-income jobs with unsafe working conditions, increasing their susceptibility to abuse and exploitation, while the trauma from the abuse can hamper their ability to secure stable jobs with better working conditions [17,18]. In some instances, risky work arrangements, such as co-location of employers and employees for some precarious job categories (e.g., care workers and domestic workers), can increase vulnerability to abuse and exploitation, illustrating the interconnectedness of precarious employment and GBV [11,19–21]. Furthermore, studies conducted on intimate partner violence (IPV) and job stability have shown how partners can employ several tactics to sabotage their partners at work [22–24]. The abuse at home can also result in reduced performance at work, frequent work absences, and lateness, which potentially trigger workplace violence concealed within harsh disciplinary actions for perceived incompetence [22,23,25]. These instances of abuse often result in women failing to maintain their jobs, having gaps in employment history, which may push them into temporary and informal jobs all characterized by lower wages, fewer to no benefits, and job precarity [7].

The prioritization of decent work in the United Nations (UN) Sustainable Development Goals (SDGs) #8 (Target 8.8: decent work and economic growth) underscores the need to advance understanding and work that addresses migrant women’s experiences in the precarious labor market and the complex relationships between precarious employment and GBV. Prioritization of decent work is crucial to achieving SDG Goals #1 (no poverty); #3 (good health and wellbeing), #5 (gender equality) and #16 (promote peaceful and inclusive societies for sustainable development), all important in mitigating GBV against migrant women in precarious employment and furthering gender equality and development goals. When decent work is not prioritized, it is difficult to eradicate poverty, achieve gender equality, and achieve good health.

The relationship between GBV and precarious employment remains underexplored with notable paucity of both empirical research and comprehensive literature reviews that critically examine the intersections of these phenomena. Two scoping reviews have been conducted by McGregor and colleagues [7,24], which focused on violence and work from a general perspective, i.e., examining the benefits and drawbacks of work in the context of IPV, and on women in general. In response to McGregor and colleagues’ [7,24] call for the need to understand the needs and experiences of understudied population groups regarding work and violence, this review focused specifically on GBV and precarious employment among migrant women. Our review was guided by the following research question: *How does the existing literature describe the interconnectedness of GBV and precarious employment among migrant women?*

### Theoretical frameworks

#### Intersectionality.

Intersectionality has been increasingly utilized in studies focusing on GBV to better understand the extent to which multiple systems of oppression and inequalities converge and exacerbate women’s vulnerability to violence [26–30]. As argued by Shannon and colleagues [31], an intersectional perspective rejects a single-axis analysis in understanding women’s GBV experiences but focuses on the overlapping processes and inequalities that reinforce GBV [32,33]. In this review, intersectionality is valuable in illuminating the convergence of various systems of oppression and inequalities that impact women’s access to precarious employment and how this shapes their experiences of GBV. Understanding the interconnectedness of precarious employment and GBV against migrant women requires looking beyond single categorizations of identities and recognizing that numerous factors intersect to shape their lived experiences [34]. Homogenization of migrant women’s experiences of GBV and precarious employment conflates within-group differences and obscures the lived realities of migrant women [35]. Thus, adopting an intersectional lens is appropriate for a comprehensive analysis that accurately captures the lived experiences of migrant women in precarious employment and at the risk of GBV.

#### Feminist political economy framework.

The Feminist Political Economy (FPE) framework, widely applied across disciplines such as economics, social work, and public health, is essential for understanding inequality and analyzing how gender, power, and class along with economic, political, and cultural forces interact to impact access to resources and opportunities [36]. The FPE framework critiques the way in which patriarchy and neoliberalism, and the substantial changes brought by neoliberal restructuring and policies, have weakened labor protections and pushed women into precarious work [37,38]. Several studies have utilized this framework to better understand labor inequities and the need for equitable policies to increase the value of work assigned to women [36]. For instance, the FPE framework has been used to assess the impacts of digital labor platforms on the regeneration of intersectional inequalities [39] and to examine women’s roles in informal and precarious work during the COVID-19 pandemic [36]. The FPE framework allowed us not to only focus on the micro-level power dynamics experienced daily by migrant women in precarious jobs such as domestic and care work, but also on macro-level influences like labor and state policies that contribute to economic and legal precarity, and shape migrant women’s experiences of abuse and exploitation [40]. Through this framework, GBV and precarious employment are not only regarded as individual issues but structural ones, exposing the gendered and racialized nature of precarious employment that places migrant women at heightened risk of abuse and exploitation.

By integrating intersectionality theory and the FPE framework, this study adopts a comprehensive approach to understanding GBV within broader economic structures. It also considers the diverse, intersecting vulnerabilities faced by migrant women. This integration enhances the study’s ability to analyze power dynamics, structural inequalities, and policy gaps, ensuring that the findings reflect both the systemic drivers of labor precarity and violence, as well as the lived experiences of migrant women.

## Methods

This review was guided by Arksey and O’Malley’s [41] five steps for conducting scoping reviews, which in broad terms, include identification of research questions, a search in relevant databases, selection of relevant articles, charting the data, and extracting relevant information from the articles and summary and report of the results. We selected a scoping review methodological approach to support a broad exploration of the literature to support the research question. This scoping review was registered on Figshare to ensure transparency and reproducibility [42]. Our reporting of methods and findings follows the PRISMA Checklist [43] and scoping review specific reporting guidelines.

### Article search

An initial search strategy was developed with the assistance of a medical librarian (S1 Table). The following electronic databases were searched: Medline, Embase, Web of Science, Scopus, EconLit, CINAHL, and PsycInfo in May 2024 and updated in May 2025 using the same search strategy. The search strategy was developed based on the following key domains from the research question: *precarious employment*, *gender-based violence,* and *migrants*. We adopted the UN definition of GBV, which is defined by the Committee on the Elimination of Discrimination Against Women (CEDAW) [44] as any harmful behaviour ‘that results in, or is likely to result in, physical, sexual or psychological harm or suffering to women, including transwomen, including threats of such acts, coercion or arbitrary deprivation of liberty, whether occurring in public or private life”. Although the definition of precarious employment remains multifaceted, we adopted the ILO [11] definition. Precarious employment is work performed in both a formal and informal economy and characterized by uncertainty in duration of employment, lack of access to social protection and benefits associated with employment, inadequate income, poor working conditions, and “lack of rights and protection due to substantial legal and practical obstacles to joining unions and bargain collectively” [11].

We also adopted the UN definition of international migrants, which pertains to individuals living (legally and illegally) in a country other than their country of birth [45]. Our focus was on both racialized and non-racialized migrant women to better understand the diversity of experiences across different migrant groups in different geographical, cultural, and socio-economic contexts. These definitions informed our search strategy, serving as operational criteria for title/abstract and full-text screening, while guiding the identification, adaptation, and modification of pertinent keywords and search terms for each database.

### Inclusion and exclusion criteria

Articles were assessed for inclusion in this study based on the following SPIDER (Sample, Phenomenon of Interest, Design, Evaluation, Research type) tool [46]. Table 1 presents the inclusion and exclusion criteria that guided study selection in this review.

**Table 1:** Inclusion and exclusion criteria according to the SPIDER tool.

Sample	Studies focusing on migrant women (including trans women) who: 1. Are aged 15 years and above. The age of 15 years is set as the lower threshold in the inclusion criteria because data collection efforts predominantly focus on women aged 15 years and above to encompass experiences of violence in contexts where early marriages are prevalent [47]. Moreover, ILO often defines the working-age population as all persons aged 15 years or older [48]. Articles addressing adult women without specifying age ranges were also included in the review. 2. Self-report engagement in precarious employment 3. Self-report experiences of GBV
**P**henomenon of **I**nterest	Experiences of GBV and engagement in precarious employment. The focus is on studies that report on the interconnectedness of precarious employment and GBV, not on GBV and PE as standalone outcomes.
**D**esign	Qualitative (e.g., focus group discussions, interviews, participant observation) or quantitative (e.g., cross sectional, cohort) and mixed methods
**E**valuation	Experiences, views, and opinions on the interconnectedness of precarious employment conditions and GBV risks and experiences among migrant women
**R**esearch Type	Peer-reviewed studies that are based on primary research and published in full-text format* with no year parameters.

*Reviews, studies published as conference proceedings, dissertations, and abstracts were excluded.

### Study selection

**Fig 1:**
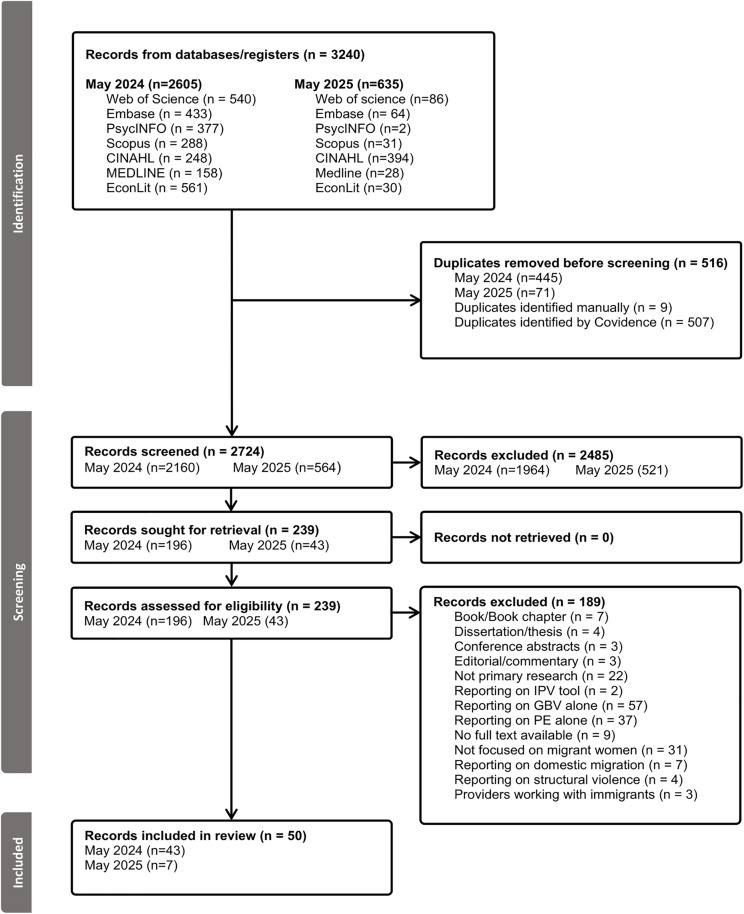
PRISMA flow diagram of the study selection process.

We utilized Covidence as our data management platform (https://www.covidence.org/), and results from the searches were uploaded for deduplication and screening. A pilot test of 20 studies was conducted by CC and NA, and afterwards four reviewers (CC, PKN, NA, RR) independently conducted title/abstract and full text screening against the inclusion and exclusion criteria to determine which articles to include for analysis. Any discrepancies were initially discussed among the reviewers, and in cases where consensus could not be reached, a third reviewer was consulted. This resolution process was conducted transparently to allow open discussion among the reviewers. The study selection process is summarized in Fig 1.

### Data extraction

Data were extracted from the included studies by two independent reviewers (CC, RR) using a data extraction form developed in Covidence. The data extraction form was pilot tested with 10% of the included studies by two reviewers (CC, NA), and the reviewers discussed whether any further modifications were required. Study elements extracted for each article included author name, publication year, country, job type, GBV type, research aim/objective, methods, key findings, and policy implications.

### Methodological quality appraisal

We did not conduct a quality assessment, considering that the objective of this scoping review is to examine the interconnectedness of precarious employment and GBV, and not consistent with scoping reviews [49].

### Data analysis

**Fig 2:**
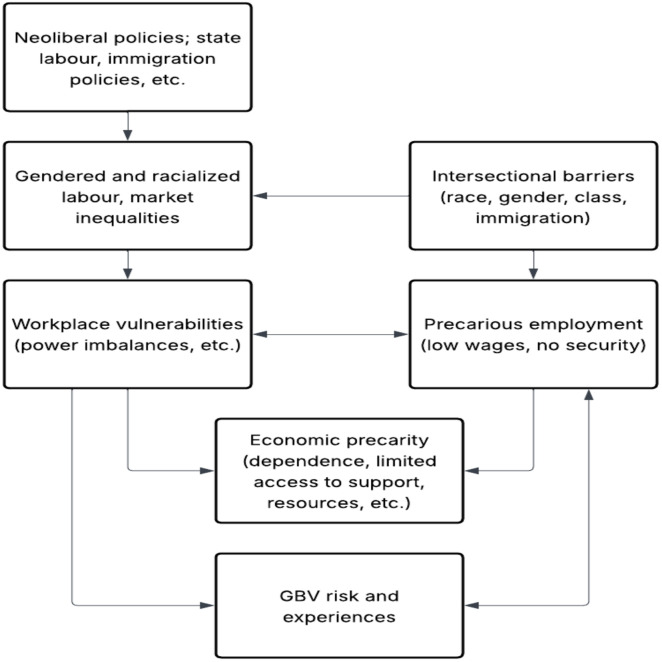
The interconnectedness of GBV and precarious employment.

Our results were synthesized through a qualitative narrative approach [50]. Data were analyzed thematically, guided by the intersectionality theory and the FPE framework. An intersectionality lens was employed to understand how various social identities such as gender, race, class, and immigration status intersects to shape migrant women’s experiences of abuse and exploitation both within and outside the work environment. We further utilized the FPE framework to examine the broader economic structures and power relations that influence these intersections. This framework enabled an analysis of how economic and labor market policies, along with the gendered and racialized nature of precarious employment and state policies, contributed to employment precarity and increased vulnerability to GBV. Our focus was on understanding the multi-directional relationships that impact how precarious workplace conditions shape or contribute to various forms of GBV risk and experiences and how the consequences of GBV influence migrant women’s engagement in precarious employment. The emerging themes were analyzed at the intersection of precarious employment and GBV. Fig 2 illustrates how precarious employment and GBV intersect, revealing how workplace or economic vulnerability not only heighten the risk to abuse but is also exacerbated by GBV experiences.

### Patient and public involvement

Neither patients nor members of the public were involved in or consulted in the development of this scoping review. However, continued consultations and engagement with academic and non-academic experts working at the intersections of precarious employment and GBV informed the conceptualization of the study, the search strategy, and the interpretations of the findings.

### Ethical statement

This scoping review did not require ethics approval as it did not involve the collection of data from human subjects.

## Results

### Characteristics of included studies

From both the initial and updated search, a total of 50 studies from 25 countries were included for analysis. No publication year limits were applied in our selection process. The studies that met our inclusion criteria were published between 2009 and 2025 (S1 Table). The majority of the studies (82%) were conducted between 2015 and 2025. Most of the studies were conducted in the Middle East, including Israel (14%), Lebanon, Yemen, Kuwait (9%), followed by the United States (US) (20%), and China, Ethiopia, Canada, and Spain (7% respectively). The majority of studies (80%) focused solely on migrant women’s experiences, while 16% examined both migrant women’s and service providers’ perspectives. Only 4% of studies specifically addressed service providers’ viewpoints. Workplace physical violence in its various forms (78%) was the dominant focus in the literature on precarious employment, while relatively few studies (22%) examined IPV/domestic violence among migrant women in precarious employment. The majority of the studies focused on domestic workers (50%), care workers (22%), farm workers (12%), and hospitality industry workers such as restaurant workers, hotel housekeepers, and cleaners (14%). Most of the studies were conducted using qualitative methods (74%), and a few used mixed methods (10%) and quantitative methods (16%). The populations of migrant women represented in the included studies are Southeast Asians (32%), Black (African) (22%), Latine/x (20%), and South Asian (8%). Few studies (18%) did not specify the race of the migrant women included in the study.

Across the included studies, migrant status was defined using a range of classifications, including terms such as undocumented, asylum seekers, permanent residents, and international students. In this review, undocumented migrant women included those whose legal status had expired and those who entered the host country illegally. The types of visas reported across studies include employer-specific work visa, spousal visa, and student visas. Half of the studies did not specify the immigration status of migrant women (50%). In some cases, this was due to migrant women refusing to disclose their status, while in others, researchers refrained from asking to avoid ethical concerns related to safety, confidentiality, and potential legal risks. However, a few studies described migrant women as either documented (32%) or undocumented (18%). Very few studies (10%) explored the perspectives of returnee migrants who had been employed in precarious employment in other countries.

Our results are reported in two sections. The first section discusses the intersections of GBV, and precarious employment and the second section discusses the emergence of human trafficking at the intersection of GBV and precarious employment.

### Intersection of GBV and precarious employment

#### Socio-cultural influences on precarious work and GBV risks.

Migration often triggers a reconfiguration of gender roles as migrant women navigate new environments in the host countries. This transition can be complex as migrant women balance their pre-migration traditional roles (e.g., unpaid caregivers in the home) rooted in patriarchal systems, with the new labor market realities that provide them with an income and often require them to work in unsafe work conditions. Across studies, migrant women reported that the intensified frustrations and violence in the home were exacerbated by the disruption of cultural and social expectations around gender roles in intimate relationships- post-migration (e.g., man as breadwinners and women as caregivers) [51–53]. The economic pressures, mostly rooted in poverty post-migration, often force women to take on paid work outside their caregiving roles within the home, resulting in a shift in family dynamics. This economic empowerment can result in IPV within the home as male partners struggle with performing their culturally expected roles as primary earners [52]. For example, in a study conducted in Australia, migrant women reported that even though having access to work and an income reduced their dependency on their partners, they also ran the risk of being forced into precarious jobs, particularly cash jobs with unsafe working conditions [54]. This was mainly because their partners (as a way of asserting control) refused to contribute to paying bills such as rent, leaving women with many financial obligations that could not be met with the salary from one job [54].

Few studies discussed how partner sabotage is not only a form of IPV but is also embedded in broader structural and systems with migrant women facing compounded challenges where gendered expectations, immigration status, and partner control intersect to restrict economic opportunities, leading to a reliance on precarious work with limited protections [54,55]. For women under spousal visas, the economic precarity that women experienced intersected with their partner’s economic control, which was grounded in threats to return to their home country of origin if women did not give their partner control over their income or comply with their sexual demands [54,55]. A study conducted in Canada highlighted how migrant women enter marriages of convenience to obtain legal immigration status for employment purposes, creating a pathway of sexual abuse and threats of deportation and termination of employment [55]. Migrant women who secured employment through their partners often faced public humiliation from both their partners and employers (who were typically friends of the partner) [55]. In another study conducted in the US with immigrant women who experienced IPV, access to transport to travel to workplaces was used by partners to block women from working by either using the car the same time its needed for work, failing to pick up or drop women on time at work and not giving women transport fare to go to work [56]. By limiting women’s access to transport, partners caused increased lateness and absenteeism at work, restricted migrant women’s job search options, and limited them to precarious jobs [56].

#### Workplace violence as a gendered and racialized experience.

Most of the studies reported on how precarious labor practices, largely characterized by exploitative working conditions, limited benefits, exacerbated by power imbalances and lack of legal protections, create a fertile ground for abuse of migrant women [53–55,57–76]. Precarious employment conditions create an environment where gendered violence is tolerated and often goes unaddressed and unreported, mostly due to precarious immigration statuses intersecting with poverty and limited awareness of migrant women’s rights [54,55,62,63,65,73,74,77,78]. For instance, a study conducted with Filipino migrant women in Israel highlighted that it was common for novice migrant home care workers to bathe their employers with their shirts off because this was presented to them as part of their job requirements, thus increasing their vulnerability to sexual abuse [79]. However, Filipino migrant women in Taiwan were reported to be generally more aware of their rights than women from Indonesia and Vietnam due to higher education levels and greater westernization influenced by their socio-economic backgrounds and cultural contexts [57].

In some studies, *quid pro quo* is used as a method of coercive control, preying on the vulnerabilities of migrant women to obtain sexual favors [55,67]. This issue is worsened by weak workplace regulations and restrictive immigration policies that label undocumented women as illegal, making it difficult for them to seek support and report abuse. For example, studies conducted in Canada and the US highlight that supervisors ask for sexual favours in exchange for better working hours, along with sexual insinuations with lips and eyes, and physical grabbing, followed by threats of job termination if women do not comply [55,63,67]. Misogyny is also evident as women reported being seen as sex objects, with gestures and conversations that undermine their purpose for going to work [55,61]. Even after changing jobs, the problem persisted with male supervisors asking for sexual favours [67]. However, women found it harder to report for fear of deportation due to their tenuous immigration status.

Importantly, there is evidence of workplace violence moving beyond the binary notions of male perpetrators and female victims, especially in feminized occupations such as domestic and care work. Wives of male employers also enforced exploitative practices and perpetrated workplace violence against migrant women. For instance, migrant domestic workers living in their employer’s residence experienced occasional physical and emotional violence and death threats, which led to suicide from wives who became jealous [74,80]. In another study conducted in Spain involving domestic and care workers, some women experienced ongoing abuse with female employers often denying them food and subjecting them to verbal abuse, while daughters in the household frequently shouted at them [75]. This persistent abuse reflects a broader pattern of gendered and racialized power dynamics within domestic labor settings.

Notably, few studies highlight how the lack of workplace protections in precarious work environments intensifies racial discrimination with supervisors and co-workers exerting control over migrant women based on racial prejudices, thereby heightening the risk of abuse and exploitation [55,59,62,63,65,68,71,74,81]. In Canada, a study involving Latine/x women in the hospitality sector revealed that race (whiteness) serves as a protective factor against sexual abuse, with migrant women facing greater exposure to sexual abuse and possessing limited power to negotiate or contest such treatment compared to non-racialized Canadian women [55]. Another study [65] emphasized the importance of using an intersectional lens to examine the experiences of Black care workers from Liberia, South Sudan, Nigeria, Zambia, and Sierra Leone employed in nursing homes within predominantly white US neighborhoods to shed light on how race and unequal social structures contribute to experiences of abuse in the work environment. In this study, migrant women reported experiencing racial discrimination, being sworn at because of their skin color, called derogatory names, and subjected to physical violence, such as having hot coffee spilled on them, with their white supervisors doing little to address or prevent workplace abuse [65]. A study conducted with Latine/x platform-based cleaners in Germany, highlighted how imbalance in power relationships with clients based on race paves way for abuse with employers often taking the side of the German white clients over the Latin American migrant female workers, leaving women highly vulnerable to persistent abuse and exploitation [82].

The intersection of gender, ethnicity, and class manifests as within-group discrimination among racialized migrant women, indicating that racial discrimination extends beyond skin color to include factors such as nationality, language, class, and socio-economic background, reflecting its deeply rooted and multifaceted nature [63,71,75,82]. In a study conducted in the US focusing on Latino migrant housekeepers, some migrant women reported experiencing compounded verbal abuse and harassment from both their supervisors, who favored African Americans, and their Latine/x colleagues, with women from Mexico emotionally abusing women from El Salvador based on ethnicity [71]. In some instances, women face institutional racism in the form of disproportionate qualification and language requirements imposed on newly arrived immigrants. For example, in a study conducted with women in domestic and care work in Spain, there is a firm requirement to remain registered in a municipality for three years and complete a Catalan language course before their residency and employment status can be formalized and these conditions increase their vulnerability to exploitative and abusive labor practices [75].

Service providers also recognized the patterns of abuse and noted how precarious work conditions created a fertile ground for the exploitation and abuse of migrant women [51,57,79]. Remarkably, recruitment agents offered a contrasting perspective, often justifying the abuse faced by migrant women in workplaces. In a study conducted in Lebanon, managers of recruitment agencies justified the physical and verbal chastisement of migrant women by their employers, arguing that such measures were necessary to control the workers and instill discipline, ensuring that duties were performed according to contract terms [83]. In some cases, recruitment agents even instructed employers to bring migrant women to their offices for disciplinary actions to ensure compliance [83]. This reflects how the absence of weak legal protection and labor policies can facilitate abuse and exploitation by creating pathways where recruiters can also abuse migrant women without any repercussions [66]. Furthermore, it also highlights the deeper issues of control, misogyny, and patriarchal norms that support the belief that women need to be controlled and disciplined, reflecting a broader societal acceptance of gender-based power imbalances and normalization of abuse.

#### Inter-relationships between financial insecurity, precarious employment and GBV.

Several studies discussed financial insecurity as an outcome of precarious employment and one of the key factors exacerbating migrant women’s risk of experiencing GBV, which encompasses a broad range of factors, including debt bondage, low wages, and familial obligations [53,58,62,66–70,72,78,79,84,85]. Notably, across several studies, financial insecurity, intersecting with factors such as poverty and country of origin, significantly impacted migrant workers by increasing their vulnerability to abuse and exploitation [71,78,86].

#### Debt bondage.

In this review, debt bondage is understood as a situation where migrant women work to pay off their debts incurred during the migration process. Across several studies, debt bondage was identified not only as a modern form of slavery but also a cornerstone of the precarity faced by migrant women and results, in part, from the large financial debt incurred by migrant women because of their premigration related costs including recruitment fees, safety deposits to prevent overstay and unauthorized work and mandatory savings imposed in receiving countries [53,58,70,72,75,79,84,86–90]. Evidently, the debt experienced is multifaceted, with migrant women being indebted to their employers, the state, their recruitment agents, and their families and friends [58]. Some women borrow money from friends, family, and employers with the promise of paying them back, while others take loans that amount to thousands of dollars to acquire work permits [67,84,88].

The lack of strong legal protections emboldens employers to use debt as a tool of coercion and abuse, allowing employers to maintain a flexible and disempowered workforce that cannot easily seek justice, contest debt agreements, or find alternative employment without risking deportation (particularly for undocumented migrant women) [58,72,87]. In Vietnam and Japan, migrant women were required by government-sanctioned practices to make a safety deposit ranging from US$6,000 to US$10,000 to prevent absconding, with contracts stipulating that they must complete their labor terms to reclaim these deposits, thereby making it extremely difficult for them to escape violence and exploitation [58]. In another study conducted with Filipino migrant women and service providers in Israel, financial insecurity as a result of debts extended throughout the government/agency level and the family level with home care workers instructed to pay agencies in the Philippines and Israel to secure employment, force migrant women to work in domestic labor under unsafe circumstances, to pay their debts [79]. Consequently, migrant women are forced to endure instances of exploitation and violence and desist from claiming their rights and reporting instances of physical and sexual abuse within their employment environment [57,58,79].

#### Low wages and the financial burden of familial obligations.

Low wages and familial obligations create a compounding financial burden for migrant women in precarious employment, deepening their vulnerability to GBV [58,70,84]. The intersecting factors of poverty, immigration status, and economic disadvantages place migrant women at the margins of society, where low-paying jobs are their only available option [88]. In several studies, migrant women were reported to enter live-in labor roles with low wages that are seemingly beneficial due to enhanced ability to meet their financial obligations (e.g., caring for their families back home), further entrenching women in cycles of financial insecurity and poverty leaving little room for escaping the abuse and the exploitative work conditions [53,68,91]. For instance, in studies with migrant domestic workers, financial insecurity, resulting from the pressure to support family back home, forced migrant workers to engage in sexual activities with their employers to get additional income [55,63,67,69].

This layered oppression highlights how migrant women can experience both GBV and economic exploitation, shaped by their unique social identities, such as socio-economic status, race, and gender. Notably, for migrant women in the US who rely on cash payments mostly due to their undocumented status, there is an increased risk of GBV, such as violent robberies and rape, mainly linked to gender rather than race [68]. Regardless, migrant women desist from reporting their experiences of abuse primarily due to the risk of losing income [53,68], thus prioritizing financial stability over leaving violent situations.

### Power imbalances in work-life dynamics

#### Power imbalances in work environments and vulnerability to GBV.

In this review, power imbalances were more pronounced among migrant women working in domestic work, care work, and informal work. Familiasm (a cultural value that emphasizes warm, close, supportive family relationships) was reported to be a key pathway for abuse of migrant women working in home environments [54,55,57,63,66–68,72,74,81,87,92]. In a study conducted in Turkey with migrant live-in domestic and care workers, familialism fostered an abusive environment for migrant women due to blurred boundaries between ‘family member’ and ‘employee’ [92]. This provides a fertile ground for manipulation, resulting in employers withholding work permits from women and increasing their workloads [92]. By treating migrant women as family members, a sense of loyalty is ingrained in them [54], forcing them to make sacrifices that expose them to vulnerable situations. In this regard, workplaces become precarious spaces where one is regarded as a family member on one end and exploited and regarded as a low-class laborer on the other end [57,92]. For migrant domestic workers from Indonesia, Vietnam, and the Philippines, the exclusion of migrant women from the family kinship (a network of family relationships that include multiple households) did not reduce or prevent abuse, as women were regarded as strangers and laborers and subjected to abuse and exploitation [57].

Very few studies reported on the phenomenon of gratitude trap, a situation where migrant women feel obligated to remain loyal and compliant to their employers even when it comes at a personal cost [54,70,91]. In a study conducted in Spain with migrant women from Peru, Colombia, Ecuador, Paraguay, Nicaragua, and Honduras, migrant women felt content with their current work situations characterized by abuse and harsh working conditions, viewing it as less precarious than their previous jobs in their home countries, and grateful for the job opportunities [91]. Likewise, migrant domestic workers in Chile regarded their employers as “good” despite the physical and emotional abuse they faced, along with food deprivation, unsafe living conditions, low wages, and long working hours [70].

#### Work-family life imbalance.

Several studies highlight how migrant women often face excessively long working hours, particularly in roles such as domestic work, home-based care, farm labor, and agriculture, which makes them more vulnerable to abuse from their employers [57,64,83,93]. In some studies, long working hours were reported to have limited migrant women’s access to social support, increase their vulnerability to abuse, and hinder their ability to pursue further training and education, making it difficult for them to integrate into the labor market [70,77,84]. Likewise, migrant workers report experiencing schedule unpredictability, which entails women showing up for work any day or night [54,77]. The unpredictability of work schedules resulted in women failing to maintain a balance between their personal/family commitments and professional/working lives, and in extreme cases, face unemployment, thus increasing their susceptibility to IPV [54]. This is more prominent among migrant women working in informal employment who are not protected through social schemes in both the country of origin and the destination country [69,94]. This highlights how work-life imbalance should not be seen as migrant women’s failure to balance responsibilities, but rather as a structural issue caused by policies and practices that undervalue women’s unpaid care work and overlook the burden of their exploitative, precarious paid jobs.

The outcomes of work-life imbalance (e.g., exhaustion and stress) were reported to significantly contribute to the presence of IPV, often resulting in migrant women and their partners struggling with cognitive tasks such as problem solving, which made it difficult for them to de-escalate any conflicts within the home [52]. A study conducted with Eritrean migrant women in Israel highlighted how economic and family pressures, along with stress and extreme exhaustion from working long hours (12–18 hours per day), make it difficult for couples to resolve their differences or seek guidance from their social networks, leading to severe IPV and femicide [69].

### Human trafficking at the intersection of GBV and precarious employment

Human trafficking, often described as a form of modern slavery involving the recruitment, transportation, harbouring and/ or exercising control, direction or influence over the movements of a person to exploit that person, typically through sexual exploitation or forced labor [95,96], was not an explicit focus of this review. However, we identified a few studies (n = 14) that discussed experiences of coercion, exploitation and vulnerability that align with the defining elements of human trafficking. This convergence of GBV, precarious employment, and human trafficking underscores profound structural and systemic inequalities in labor and economic protections, gendered labor dynamics, and restrictive immigration policies, which, combined with social identities like race, gender, and immigration status, significantly increase the vulnerability of migrant women [58,60,66,72,73,75,79,85,86,89,90,93,97,98].

Across studies, debt bondage and social isolation, including confiscation of passports and deception in recruitment characterized by fake contracts and fake visas both in the country of origin and the receiving country, were reported to lay the foundation for abuse, coercion, forced labor, and sex trafficking [58,73,80,85,90]. In a study conducted in the US, threats that involved physical or sexual assault in the workplace and restrictions on undocumented migrant women’s physical or communicative freedom, particularly for those working in domestic and care work, were counted as elements of labor and sex trafficking [60]. Restricting communication often forced migrant workers to be dependent on acquaintances made in the host country, thus placing women at risk of immersing themselves in networks involved in human trafficking [66]. Across several studies, women were restricted from speaking on the phone and watched through cameras, limiting their mobility and communication with social networks, thus mimicking modern-day slave practices [79,85,92].

Engaging in precarious employment and the informal labor market is not an individual choice. Labor sponsorship policies, like the *kafala* system (labor sponsorship scheme, more like a guest work programme in which migrants are not legally permitted to settle in the host country), in the United Arab Emirates prevent migrants from migrating into the formal sectors of labor after legally settling in the host country [69,86,90]. Policies like this make it easier for migrants to become illegal and undocumented, thus introducing the likelihood of irregular and exploitative migratory patterns, where women are often abused by employers and face restrictions on their mobility and income-earning potential. In some instances, migrant women may end up pursuing the informal sphere of sex work in pursuit of additional income, thus increasing their likelihood of being trafficked [78,86,90].

Additionally, human trafficking can be embedded in the recruitment process, with some migrant women reporting that they refused offers from recruitment agents for informal employment due to its association with sex trafficking [87]. In a study conducted with returnee migrant women in Sri Lanka who had worked in Saudi Arabia, Kuwait, the United Arab Emirates, Lebanon and Jordan, migrant women who were recruited as domestic workers were also forced into commercial sex upon arrival or expected to be sex slaves to their masters while still performing their domestic worker duties [85]. Ethiopian returnee migrants who had worked as live-in domestic workers in Yemen, Kuwait, Saudi Arabia, and Bahrain were forced to be sex slaves to their masters while still performing their domestic work, and did not report for fear of deportation [80]. In a study involving domestic workers in China, it was found that some women only discovered upon arrival that their roles extended beyond domestic work to include coerced sexual servitude to the husband, often with the covert approval of both the spouse and the mother [98]. Women who resisted this exploitation and attempted to escape were sometimes returned to recruitment agents, who then placed them with their own families to work for extended periods without pay, reflecting clear elements of labor trafficking [98].

While not explicitly focused on human trafficking, a study conducted in Canada with service providers and decision-makers highlighted that debt bondage and social isolation were significant factors pushing women to remain silent and endure abuse and exploitation [73]. These conditions were described as creating a form of captivity under their employment contracts [73], thereby increasing the risk of human trafficking.

## Discussion

Our review critically examined the interconnectedness of precarious employment and GBV among migrant women, extending the scant literature on this topic. As this scoping review reveals, there is paucity in the current state of knowledge regarding the intersection of GBV and precarious employment. With 50 relevant studies globally from 2009 to 2025, the body of knowledge is not commensurate with the magnitude of this issue, given the significant rise of migration, precarious work, and GBV globally [99]. However, the majority of the studies were conducted post-2015, which indicates the growing interest in this topic, in line with SDGs #5 and #8, which primarily focus on gender equality and decent work. By centering our analysis on the interconnectedness of GBV and precarious employment through an FPE framework and an intersectional lens, this paper demonstrates how migrant women are at the risk of abuse and exploitation not only because of individual factors but also social and systemic structures along with state policies that shape their experiences of violence and labor opportunities.

Our review underscores the structural and institutional dimensions of precarious employment and GBV, which extend beyond individual acts of abuse to broader systems of labor market discrimination, social exclusion, and policy neglect that place women at the risk of exploitation and abuse. The structural conditions of precarious employment not only contribute to abuse and exploitation but also restrict pathways to exit, mostly due to fear of job loss or migration-related repercussions, particularly for undocumented migrant women. Thus, failure to acknowledge the contribution of precarious employment in GBV experiences and risks may perpetuate the normalization of economic exploitation, violence within and outside workplaces, and gendered discrimination, leaving migrant women with limited pathways for protection or justice.

Precarious employment is often seen as an economic issue, but its gendered dimensions reveal a deep connection to GBV that remains largely unaddressed. The gendered nature of precarious employment results in migrant women being disproportionately concentrated in precarious work, as highlighted in our review, with most migrant women involved in domestic labor, care work, and hospitality work. Our findings further underscore the persistent gender-based stereotyping and discrimination faced by migrant women in the labor market, particularly those employed as care workers and domestic workers. Contrary to previous studies [17], our research reveals that precarious work, characterized by part-time and irregular contracts, does not fully benefit migrant women with caregiving responsibilities; instead, the inflexibility of these contracts and unpredictable schedules exacerbate work-life imbalance. This imbalance is more of a structural issue than an individual shortcoming, as the uneven burden of reproductive work, aligned with patriarchal expectations, makes it challenging for women to balance work demands, thereby increasing their risk of GBV within and outside the workplace.

Our findings further highlight the compounded disadvantages faced by migrant women in precarious employment due to intersecting systems of oppression, particularly those based on race and gender, which increase vulnerability to GBV. The dual impact of racial discrimination and misogyny creates a complex web of vulnerabilities, making migrant women at an increased risk of abuse. Contrary to previous studies [100,101],our findings also reveal how race can act as either a protective or risk factor in the perpetration of GBV against migrant women in precarious employment, highlighting the dual role that race plays in GBV risk and experiences. Additionally, the existing evidence underscores the presence of misogyny in the abuse of women in precarious workplaces, with the sexual objectification of migrant women’s bodies often leading to instances of sexual abuse and harassment from employers. This recurring pattern reveals the risky nature of precarious employment, highlighting how misogyny and racial discrimination perpetuate the cycle of abuse in precarious workplaces.

Further, the intersection of race, gender, and immigration status with pre-migration factors such as poverty further amplifies the risk of exploitation and abuse, with migrant women facing unique vulnerabilities to both their employers and partners. Similar to previous studies on pre- and post-migration concerns for migrant women [102], our findings showed that pre-migration factors, particularly poverty, force women into precarious migration pathways, which often involve accruing financial debt to fund the migration processes. Upon arrival in host countries, these vulnerabilities are exacerbated by factors such as racial discrimination, economic insecurity, and restrictive immigration and labor policies, further entrenching migrant women in poverty and repeated abuse and exploitation. As a result, migrant women endure abuse and exploitation, with little recourse due to the fear of job loss and income loss, deportation, or further economic precarity.

In this review, the indicators of precarious employment, GBV, and human trafficking are clearly illustrated through deceptive recruitment practices such as fake visas and work contracts, debt bondage, restrictions on movement and communication, isolation, and exploitative and abusive conditions. However, contrary to previous studies [99], our findings go a step further by highlighting a significant yet often overlooked dimension of precarious work, GBV and human trafficking, which is exacerbated by restrictive policies that criminalize undocumented migrant women, leaving them with limited pathways to protection and justice. At the intersection of precarious employment, GBV, and human trafficking, our research demonstrates how migrant women frequently assume dual roles as both precarious workers and sex slaves for the same employer. In other instances, they may resort to commercial sexual exploitation to supplement their income to pay off debts and support their families. This dual exploitation underscores the severe vulnerabilities faced by migrant women in precarious employment that increase their susceptibility to GBV risk.

### Implications for policy and practice

The findings from this review have several important implications for policy and practice. First, as discussed in this review, the intersection of GBV and precarious employment among migrant women raises questions of how workplace regulations and GBV, labor, and immigration policies can be revised to ensure that migrant women in precarious employment and are at the risk of GBV are protected. Given that public policies that fail to incorporate an intersectional lens have been critiqued for multiply marginalizing migrant women [103], it is essential to integrate such a lens into existing protocols and policies on workplace abuse, GBV, and labor practices. This integration would enhance the effectiveness of current policies and programs by emphasizing multiple channels for migrant women to seek care and support, clearly stating penalties for violations and abuses by employers. Furthermore, policy measures could also include stronger enforcement mechanisms that hold employers accountable for the abuse and exploitation of migrant women as targeted and regulatory policing can play a significant role in reducing exploitation and abuse. However, given the evidence of GBV against migrant women within law enforcement agencies [104] such intervention should be carefully designed to avoid secondary victimization.

Additionally, providing further training across various industries on respecting the rights of migrant women and openly declaring that abuse and exploitation will not be tolerated is crucial. Policy makers and decision makers can also ensure that all precarious jobs have basic minimum work supports and protections, including paid family and medical leave, average working hours as stipulated by the law, right to bargain for a flexible schedule without threats of dismissal, paid sick days and job protections for all precarious workers including migrant women. Effective implementation of these reforms will require ongoing monitoring and evaluation to assess their impact.

Service providers can play a significant role by educating migrant women about their physical and psychological responses to exploitative work environments and instances of abuse, as well as the coping strategies they can adopt. As such, interdisciplinary collaboration is vital, and service providers, particularly healthcare providers and social service workers, should be prepared to make referrals for treatment and counseling and alternative housing or employment, if there is need, following the disclosure of psychological distress, abuse, and exploitation.

### Strengths and limitations of the review

We acknowledge several potential limitations in this review. Given the need to balance the scope of the review with the available personnel, we did not search grey literature. For this reason, we might have missed relevant studies that would fit our inclusion criteria. Although our review does not have language parameters, our search strategy was developed in English, hence, there is a possibility that we might have missed relevant studies that are indexed in other languages. Despite its limitations, this review has several strengths. One of the strengths of this scoping review is its breadth and inclusiveness. We conducted a comprehensive search for empirical research, including qualitative, quantitative, and mixed methods studies, focusing on all forms of GBV and all types of migrant women in various precarious jobs, without geographic or racial limitations. This resulted in a comprehensive review of the existing literature on GBV and precarious employment. This review also amplifies the voices of migrant women, highlighting their experiences at the intersection of GBV and precarious employment, an area that has previously received limited research attention.

### Key areas for further research

While this review focused solely on migrant women, it is important to recognize that the risks of GBV and precarious employment also affect other migrant groups, including men and gender diverse individuals. Therefore, further exploration of their experiences of abuse and exploitation in precarious workplaces is necessary to gain a deeper understanding of the diverse experiences and needs of all migrant workers in precarious employment and exposed to GBV. Additionally, our study highlights a close connection between GBV, precarious employment, and human trafficking. Given the interconnectedness of these issues, further investigation is required to better understand how precarious work conditions and GBV risk factors can contribute to the risk of human trafficking

Additionally, research focusing exclusively on migrant women’s insertion into the labor market, considering the different actors involved, is necessary to gain an in-depth understanding of the realities faced by migrant women and their employers in relation to the interconnectedness of GBV and precarious employment. Lastly, based on the results obtained in this research and the ongoing work of the lead author on this topic, creating practical and applicable proposals to improve labor market inclusion and the professional development of migrant women, along with recommendations for mitigating GBV, is considered a future research priority

## Conclusion

This paper focused on the interconnectedness of GBV and precarious employment among migrant women and add to the limited yet growing body of knowledge on precarious work and GBV among migrant populations. This review highlights how the intersection of precarious employment and GBV reflects deep-seated structural inequalities rooted in economic policies, gendered dynamics, and restrictive immigration systems, along with layered social identities, such as race, class, and gender, that create and sustain precarious work conditions that expose migrant women to multiple forms of GBV. Addressing the impacts of precarious employment and GBV among migrant women demands solutions that go beyond individualized support to challenge the economic and social systems that facilitate the abuse and exploitation of migrant women. This may include strengthening labor protection, revised immigration policies, and expanding social supports, ensuring workplace regulations include anti-violence initiatives. By centering the lived experiences of migrant women through an intersectional lens, we can envision meaningful interventions and policies that dismantle the root causes of labor precarity and GBV and promote the health and well-being of migrant women.

## Supporting information

S1 Table Medline search strategy. (DOCX)

## References

[1] Statistics Canada. Intimate partner violence in Canada. Daily. 2016;11:1–4.

[2] Klugman J. Gender Based Violence and the Law. Washington, DC. 2017. https://openknowledge.worldbank.org/handle/10986/26198

[3] Okeke-Ihejirika P, Yohani S, Muster J, Ndem A, Chambers T, Pow V. A Scoping Review on Intimate Partner Violence in Canada’s Immigrant Communities. Trauma Violence Abuse. 2020;21(4):788–810. doi: 10.1177/1524838018789156 30176768

[4] Kvart S, Jonsson J, Bodin T, Håkansta C, Kreshpaj B, Orellana C, et al. Precarious Employment and Psychosocial Hazards: A Cross-Sectional Study in Stockholm County. Int J Environ Res Public Health. 2021;18(21):11218. doi: 10.3390/ijerph182111218 34769737 PMC8582981

[5] Premji DS, Shakya DY. Precarious work experiences of racialized immigrant women in Toronto: A community-based study. Journal of Immigrant & Refugee Studies. 2023;5(1):1–20.

[6] Premji S. “It’s Totally Destroyed Our Life”: Exploring the Pathways and Mechanisms Between Precarious Employment and Health and Well-being Among Immigrant Men and Women in Toronto. Int J Health Serv. 2018;48(1):106–27. doi: 10.1177/0020731417730011 28906167

[7] MacGregor JCD, Naeemzadah N, Oliver CL, Javan T, MacQuarrie BJ, Wathen CN. Women’s Experiences of the Intersections of Work and Intimate Partner Violence: A Review of Qualitative Research. Trauma Violence Abuse. 2022;23(1):224–40. 32662354 10.1177/1524838020933861

[8] Benach J, Vives A, Amable M, Vanroelen C, Tarafa G, Muntaner C. Precarious employment: understanding an emerging social determinant of health. Annu Rev Public Health. 2014;35:229–53. doi: 10.1146/annurev-publhealth-032013-182500 24641559

[9] Campbell I, Price R. Precarious work and precarious workers: towards an improved conceptualisation. Econ Labour Relat Rev. 2016;27(3):314–32.

[10] Kreshpaj B, Orellana C, Burström B, Davis L, Hemmingsson T, Johansson G, et al. What is precarious employment? A systematic review of definitions and operationalizations from quantitative and qualitative studies. Scand J Work Environ Health. 2020;46(3):235–47. doi: 10.5271/sjweh.3875 31901944

[11] International Labour Organization. From precarious work to decent work: outcome document to the workers’ symposium on policies and regulations to combat precarious employment. 2012. http://www.ilo.org/wcmsp5/groups/public/---ed_dialogue/---actrav/documents/meetingdocument/wcms_179787.pdf

[12] I. L. O. ILO. Women and men in the informal economy: A statistical picture. 2018. https://www.wiego.org/wp-content/uploads/2019/09/Women%20and%20Men%20in%20the%20Informal%20Economy%203rd%20Edition%202018.pdf

[13] Ball JA. Feminization of the Labor Force, Development, and Economic Reform: Effects on Job Segregation by Sex. jda. 2008;42(1):53–67. doi: 10.1353/jda.0.0021

[14] Drolet M, Amini MM. Intersectional perspective on the Canadian gender wage gap. 2023.

[15] Daly A, Carey RN, Darcey E, Chih H, LaMontagne AD, Milner A, et al. Workplace psychosocial stressors experienced by migrant workers in Australia: A cross-sectional study. PLoS One. 2018;13(9):e0203998. doi: 10.1371/journal.pone.0203998 30235255 PMC6147467

[16] Mishra S. Feminization of poverty and dimension of women’s agencies. Asian J Multidiscip Stud. 2018;6.

[17] Paraskevopoulou A. Gender and Precarious Work. Handbook of Labor, Human Resources and Population Economics. Springer International Publishing; 2020. p. 1–18. doi: 10.1007/978-3-319-57365-6_30-1

[18] Scott A. Financial Abuse in a Banking Context: Why and How Financial Institutions can Respond. J Bus Ethics. 2023;:1–16. doi: 10.1007/s10551-023-05460-7 37359798 PMC10235837

[19] International Labour Organization. Violence and harassment in the world of work. International Labour Organization; 2021. https://www.ilo.org/wcmsp5/groups/public/---dgreports/---gender/documents/publication/wcms_814507.pdf

[20] World Bank. Migration and Poverty: Toward Better Opportunities for the Poor. 2011.

[21] Melo CL. The feminization of poverty: A critical analysis. Witn Can J Crit Nurs Discourse. 2019;1(1):73–81.

[22] Adams AE, Tolman RM, Bybee D, Sullivan CM, Kennedy AC. The impact of intimate partner violence on low-income women’s economic well-being: the mediating role of job stability. Violence Against Women. 2012;18(12):1345–67. doi: 10.1177/1077801212474294 23419274

[23] Showalter K. Women’s employment and domestic violence: A review of the literature. Aggression and Violent Behavior. 2016;31:37–47. doi: 10.1016/j.avb.2016.06.017

[24] MacGregor JCD, Oliver CL, MacQuarrie BJ, Wathen CN. Intimate Partner Violence and Work: A Scoping Review of Published Research. Trauma Violence Abuse. 2021;22(4):717–27. 31615345 10.1177/1524838019881746

[25] Asencios-Gonzalez ZB, Vara-Horna AA, McBride J. Intimate partner violence against women and labor productivity: The mediating role of morbidity. Violence Against Women. 2023. doi: 10.1177/10778012231163572 36950730

[26] Duhaney P. Contextualizing the Experiences of Black Women Arrested for Intimate Partner Violence in Canada. J Interpers Violence. 2022;37(21–22):NP21189–216. doi: 10.1177/08862605211056723 34865540 PMC9554381

[27] Warrier S. Inclusion and Exclusion: Intersectionality and Gender-Based Violence. Handbook of Interpersonal Violence and Abuse Across the Lifespan. Springer International Publishing. 2021. p. 2539–52. doi: 10.1007/978-3-319-89999-2_49

[28] Subirana-Malaret M, Gahagan J, Parker R. Intersectionality and sex and gender-based analyses as promising approaches in addressing intimate partner violence treatment programs among LGBT couples: A scoping review. Cogent Soc Sci. 2019;5(1):1644982.

[29] Kelly UA. Theories of intimate partner violence: from blaming the victim to acting against injustice: intersectionality as an analytic framework. ANS Adv Nurs Sci. 2011;34(3):E29-51. doi: 10.1097/ANS.0b013e3182272388 21822068

[30] Cardenas I. Advancing intersectionality approaches in intimate partner violence research: a social justice approach. J Ethn Cult Divers Soc Work. 2023;32(1):1–11.

[31] Shannon G, Morgan R, Zeinali Z, Brady L, Couto MT, Devakumar D, et al. Intersectional insights into racism and health: not just a question of identity. Lancet. 2022;400(10368):2125–36. doi: 10.1016/S0140-6736(22)02304-2 36502850

[32] Liu J. The precarious nature of work in the context of Canadian immigration: An intersectional analysis. Can Ethn Stud. 2019;51(2):169–85.

[33] Natalia Rocha L, Moira C, Cynthia F. Untangling multiple inequalities: intersectionality, work and globalisation. Work Organisation, Labour and Globalisation. 2015;9(2). doi: 10.13169/workorgalaboglob.9.2.0007

[34] Hankivsky O, Christoffersen A. Intersectionality and the determinants of health: a Canadian perspective. Critical Public Health. 2008;18(3):271–83. doi: 10.1080/09581590802294296

[35] Etherington C, Baker L. From “Buzzword” to Best Practice: Applying Intersectionality to Children Exposed to Intimate Partner Violence. Trauma Violence Abuse. 2018;19(1):58–75. doi: 10.1177/1524838016631128 26951190

[36] Lokot M, Bhatia A. Unequal and Invisible: A Feminist Political Economy Approach to Valuing Women’s Care Labor in the COVID-19 Response. Front Sociol. 2020;5:588279. doi: 10.3389/fsoc.2020.588279 33869516 PMC8022460

[37] Vosko LF. A New Approach to Regulating Temporary Agency Work in Ontario or Back to the Future?. ri. 2011;65(4):632–53. doi: 10.7202/045589ar

[38] Vosko L, Zukewich N, Cranford C. Precarious jobs: A new typology of employment. Perspectives on Labour and Income. 2003;4(10).

[39] Rodríguez-Modroño P, Agenjo-Calderón A, López-Igual P. A feminist political economic analysis of platform capitalism in the care sector. Rev Radic Polit Econ. 2023;55(4):629–38.

[40] Smith J, Davies SE, Feng H, Gan CCR, Grépin KA, Harman S. More than a public health crisis: A feminist political economic analysis of COVID-19. Glob Public Health. 2021;16(8–9):1364–80. 33705248 10.1080/17441692.2021.1896765

[41] Arksey H, O’Malley L. Scoping studies: towards a methodological framework. Int J Soc Res Methodol Theory Pract. 2005;8(1):19–32.

[42] Chadambuka C, Arora N, Namyalo PK, Kouyoumdjian F, Essue BM. Precarious employment and gender-based violence against migrant women: a scoping review mapping the intersections. 2024. 10.1371/journal.pone.0337690 PMC12668559 41325405

[43] Tricco AC, Lillie E, Zarin W, O’Brien KK, Colquhoun H, Levac D, et al. PRISMA Extension for Scoping Reviews (PRISMA-ScR): Checklist and Explanation. Ann Intern Med. 2018;169(7):467–73. 30178033 10.7326/M18-0850

[44] Declaration on the Elimination of Violence against Women. [Cited 2023 November 15]. https://www.ohchr.org/en/instruments-mechanisms/instruments/declaration-elimination-violence-against-women

[45] United Nations D of E and SA Population Division. International Migration Report 2015: Highlights. 2016.

[46] Cooke A, Smith D, Booth A. Beyond PICO: the SPIDER tool for qualitative evidence synthesis. Qual Health Res. 2012;22(10):1435–43. doi: 10.1177/1049732312452938 22829486

[47] Ellsberg M, Heise L. Researching violence against women.

[48] Labour Force Statistics (LFS, STLFS, RURBAN databases). ILOSTAT . [Cited 2025 March 4]. https://ilostat.ilo.org/methods/concepts-and-definitions/description-labour-force-statistics/

[49] Pham MT, Rajić A, Greig JD, Sargeant JM, Papadopoulos A, McEwen SA. A scoping review of scoping reviews: advancing the approach and enhancing the consistency. Res Synth Methods. 2014;5(4):371–85. doi: 10.1002/jrsm.1123 26052958 PMC4491356

[50] Popenoe R, Langius-Eklöf A, Stenwall E, Jervaeus A. A practical guide to data analysis in general literature reviews. Nord J Nurs Res. 2021;41(4):175–86.

[51] Priebe Rocha L, Soares C, McGregor A, Chen S, Kaplan A, Rose R, et al. Understanding Health Priorities, Behaviors, and Service Utilization Among Brazilian Immigrant Women: Implications for Designing Community-Based Interventions. J Racial Ethn Health Disparities. 2022;9(1):135–45. doi: 10.1007/s40615-020-00936-y 33403650 PMC7785287

[52] Duke MR, Cunradi CB. Measuring intimate partner violence among male and female farmworkers in San Diego County, CA. Cultur Divers Ethnic Minor Psychol. 2011;17(1):59–67. doi: 10.1037/a0021826 21341898

[53] Chung RYN, Mak JKL. Physical and mental health of live-in female migrant domestic workers: A randomly sampled survey in Hong Kong. Am Behav Sci. 2020;64(6):802–22.

[54] Vasil S. I came here, and it got worse day by day: Examining the intersections between migrant precarity and family violence among women with insecure migration status in Australia. Violence Against Women. 2023. 10.1177/10778012231159414 PMC11292973 36913733

[55] Villegas PE. “I made myself small like a cat and ran away”: Workplace sexual harassment, precarious immigration status and legal violence. J Gend Stud. 2019;28(6):674–86.

[56] Nahar S, Cronley C. Transportation barriers among immigrant women experiencing intimate partner violence. Transp Res Rec. 2021;2675(9):861–9.

[57] Pan S-M, Yang J-T. Outsiders in the Family: Abuse of Migrant Domestic Workers in Taiwan. Asian Journal of Women’s Studies. 2012;18(1):87–117. doi: 10.1080/12259276.2012.11666123

[58] Belanger D. Labor migration and trafficking among Vietnamese migrants in Asia. Ann Am Acad Pol Soc Sci. 2014;653:87–106.

[59] Zahreddine N, Hady RT, Chammai R, Kazour F, Hachem D, Richa S. Psychiatric morbidity, phenomenology and management in hospitalized female foreign domestic workers in Lebanon. Community Ment Health J. 2014;50(5):619–28. doi: 10.1007/s10597-013-9682-7 24370752

[60] Zhang SX, Spiller MW, Finch BK, Qin Y. Estimating labor trafficking among unauthorized migrant workers in San Diego. Ann Am Acad Pol Soc Sci. 2014;653:65–86.

[61] Murphy J, Samples J, Morales M, Shadbeh N. “They Talk Like That, But We Keep Working”: Sexual Harassment and Sexual Assault Experiences Among Mexican Indigenous Farmworker Women in Oregon. J Immigr Minor Health. 2015;17(6):1834–9. doi: 10.1007/s10903-014-9992-z 24514945 PMC4128901

[62] Green O, Ayalon L. Whom Do Migrant Home Care Workers Contact in the Case of Work-Related Abuse? An Exploratory Study of Help-Seeking Behaviors. J Interpers Violence. 2016;31(19):3236–56. doi: 10.1177/0886260515584347 25985975

[63] Rodríguez-Martínez P, Cuenca-Piqueras C. Interactions Between Direct and Structural Violence in Sexual Harassment Against Spanish and Unauthorized Migrant Women. Arch Sex Behav. 2019;48(2):577–88. doi: 10.1007/s10508-018-1265-9 30291600

[64] Lai Y, Fong E. Work-related aggression in home-based working environment: experiences of migrant domestic workers in Hong Kong. Am Behav Sci. 2020;64(6):722–39.

[65] Covington-Ward Y. Bodily burdens: physical abuse, workplace injury, and understanding intersectionality through the experiences of African immigrant direct care health workers. Transform Anthropol. 2021;29(2):115–26.

[66] Kouta C, Pithara C, Apostolidou Z, Zobnina A, Christodoulou J, Papadakaki M, et al. A Qualitative Study of Female Migrant Domestic Workers’ Experiences of and Responses to Work-Based Sexual Violence in Cyprus. Sexes. 2021;2(3):315–30. doi: 10.3390/sexes2030025

[67] Kim E, Hogge I. Intimate Partner Violence among Asian Indian Women in the United States: Recognition of Abuse and Help-Seeking Attitudes. International Journal of Mental Health. 2015;44(3):200–14. doi: 10.1080/00207411.2015.1035073

[68] Bevilacqua KG, Arciniegas S, Page K, Steinberg AK, Stellmann J, Flores-Miller A, et al. Contexts of violence victimization and service-seeking among Latino/a/x immigrant adults in Maryland and the District of Columbia: A qualitative study. J Migr Health. 2022;7:100142. doi: 10.1016/j.jmh.2022.100142 36568828 PMC9772540

[69] Gebreyesus T, Sultan Z, Ghebrezghiabher HM, Tol WA, Winch PJ, Davidovitch N, et al. Life on the margins: the experiences of sexual violence and exploitation among Eritrean asylum-seeking women in Israel. BMC Womens Health. 2018;18(1):135. doi: 10.1186/s12905-018-0624-y 30089494 PMC6083583

[70] Chan C, Trahms C. Managing the long-term effects of psychological abuse on (im)migrant domestic workers. Cult Med Psychiatry. 2023;dt5:7707467. 10.1007/s11013-023-09836-2 37715892

[71] Hsieh Y-CJ, Sönmez S, Apostolopoulos Y, Lemke MK. Perceived workplace mistreatment: Case of Latina hotel housekeepers. Work. 2017;56(1):55–65. doi: 10.3233/WOR-162467 28128780

[72] Bhuyan R, Valmadrid L, Panlaqui EL, Pendon NL, Juan P. Responding to the Structural Violence of Migrant Domestic Work: Insights from Participatory Action Research with Migrant Caregivers in Canada. J Fam Viol. 2018;33(8):613–27. doi: 10.1007/s10896-018-9988-x

[73] Robillard C, McLaughlin J, Cole D, Vasilevska B, Gendron R. Caught in the Same Webs - Service Providers’ Insights on Gender-Based and Structural Violence Among Female Temporary Foreign Workers in Canada. J Int Migr Integr. 2018;19(3):583–606.

[74] Diab JL, Yimer B, Birhanu T, Kitoko A, Gidey A, Ankrah F. The gender dimensions of sexual violence against migrant domestic workers in post-2019 Lebanon. Front Sociol. 2023;7:1091957. doi: 10.3389/fsoc.2022.1091957 36741584 PMC9891457

[75] Fuentes-Pumarola C, Albertín-Carbó P, Acién-González E, Sibila-Pérez M. The spiral of violence experienced by immigrant domestic workers: A qualitative approach. Violence Against Women. 2025. doi: 10778012251329264 10.1177/10778012251329263 40101280

[76] Chuemchit M, Linn N, Han CPP, Lynn Z, Chernkwanma S, Taneepanichskul N, et al. Discrimination and violence against women migrant workers in Thailand during the COVID-19 pandemic: A mixed-methods study. PLoS One. 2024;19(5):e0300388. doi: 10.1371/journal.pone.0300388 38701061 PMC11068168

[77] Ortega-de-Mora F, Terrón-Caro T. Access to labor market and integration of Moroccan women in Andalusia: The two sides of the coin. Soc Sci-BASEL. 2023;12(10).

[78] Mutambara VM, Naidu M. Negotiating in(security): agency and adaptation among Zimbabwean migrant women working in the informal sector in South Africa. Orient Anthr. 2023;23(1):54–70.

[79] Ayalon L. Evaluating the working conditions and exposure to abuse of Filipino home care workers in Israel: characteristics and clinical correlates. Int Psychogeriatr. 2009;21(1):40–9. doi: 10.1017/S1041610208008090 19040784

[80] Reda KT. The agonies and glories of female domestic workers in the Gulf states: Experiences of ex-migrants in Northern Ethiopia. Etud Popul Afr. 2015;29(2):1774–84.

[81] Gillespie A, Seff I, Caron C, Maglietti MM, Erskine D, Poulton C, et al. “The pandemic made us stop and think about who we are and what we want:” Using intersectionality to understand migrant and refugee women’s experiences of gender-based violence during COVID-19. BMC Public Health. 2022;22(1):1469. doi: 10.1186/s12889-022-13866-7 35915413 PMC9342942

[82] Mondon-Navazo M, Murgia A. It’s labour exploitation, but fortunately there is a lot of solidarity. A qualitative case study with cleaning platform workers in Berlin. Globalizations. 2025:1–19.

[83] Ghaddar A, Khandaqji S, Ghattas J. Justifying abuse of women migrant domestic workers in Lebanon: the opinion of recruitment agencies. Gac Sanit. 2020;34(5):493–9. doi: 10.1016/j.gaceta.2018.11.001 30594331

[84] Anbesse B, Hanlon C, Alem A, Packer S, Whitley R. Migration and mental health: a study of low-income Ethiopian women working in Middle Eastern countries. Int J Soc Psychiatry. 2009;55(6):557–68. doi: 10.1177/0020764008096704 19592428

[85] Wickramage K, De Silva M, Peiris S. Patterns of abuse amongst Sri Lankan women returning home after working as domestic maids in the Middle East: An exploratory study of medico-legal referrals. J Forensic Leg Med. 2017;45:1–6. doi: 10.1016/j.jflm.2016.11.001 27846452

[86] Mahdavi P. Gender, labour and the law: the nexus of domestic work, human trafficking and the informal economy in the United Arab Emirates. Global Networks. 2013;13(4):425–40. doi: 10.1111/glob.12010

[87] Kodoth P. Structural Violence against Emigrant Domestic Workers and Survival in the Middle East: The Effects of Indian Emigration Policy. Journal of Interdisciplinary Economics. 2016;28(1):83–106. doi: 10.1177/0260107915609824

[88] Green O, Ayalon L. The contribution of working conditions and care recipient characteristics to work-related abuse and exploitation of migrant home care workers. Empl Relat. 2017;39(7):1001–14.

[89] Boucher AK. Migrant sexual precarity through the lens of workplace litigation. Gender Work & Organization. 2024;32(1):458–72. doi: 10.1111/gwao.13160

[90] Trần AN. Bilateral Labor Agreement with Gendered and Unfree Labor: Vietnamese Women Domestic Workers in Saudi Arabia. J Labor Soc. 2023;27(3):370–96. doi: 10.1163/24714607-bja10123

[91] Duque T, Aceros JC, Paloma V. Sociopolitical development of female migrant domestic workers in Southern Spain: A qualitative study of a pathway against injustice. Community & Applied Soc Psy. 2022;33(2):454–68. doi: 10.1002/casp.2613

[92] Tuncer E, Eren-Benlisoy Z. Secluded lives: Restricted urban practices of migrant domestic and care workers in Istanbul. Disegnarecon. 2022;15(28).

[93] Serrano S, Martin D. Domestic violence and the health of Bolivian migrant women residents of home-based sweatshops in Greater São Paulo. Rev Interdiscip Mobilidade Humana. 2022;30(66):207–26.

[94] Cámbara FB. COVID-19 and women migrant workers in informal employment: recommendations for strengthening social protection efforts in Lao People’s Democratic Republic. Gend Dev. 2022;30(1–2):97–113.

[95] Government of Canada D of J. What is human trafficking? 2001. [Cited 2025 September 23]. https://www.justice.gc.ca/eng/cj-jp/tp/what-quoi.html

[96] Protocol to Prevent, Suppress and Punish Trafficking in Persons Especially Women and Children, Supplementing the United Nations Convention Against Transnational Organized Crime. [Cited 2025 September 29]. https://www.ohchr.org/en/instruments-mechanisms/instruments/protocol-prevent-suppress-and-punish-trafficking-persons

[97] Busza J, Teferra S, Omer S, Zimmerman C. Learning from returnee Ethiopian migrant domestic workers: a qualitative assessment to reduce the risk of human trafficking. Global Health. 2017;13(1):71. doi: 10.1186/s12992-017-0293-x 28893298 PMC5594517

[98] Ladegaard HJ. Trauma, Extreme Humiliation, and Coping Strategies in Migrant Domestic Workers’ Storytelling: Linguistic and Psychological Perspectives. J Lang Soc Psychol. 2025 Mar 25;0261927X251326290.

[99] Lewis H, Waite L. Asylum, immigration restrictions and exploitation: Hyper-precarity as a lens for understanding and tackling forced labour. Anti-Traffick Rev. 2015;5.

[100] Sabri B, St. Vil NM, Campbell JC, Fitzgerald S, Kub J, Agnew J. Racial and Ethnic Differences in Factors Related to Workplace Violence Victimization. West J Nurs Res. 2015 Feb 1;37(2):180–96. 24658287 10.1177/0193945914527177 PMC4169764

[101] Freedman J, Sahraoui N, Tastsoglou E. Thinking about Gender and Violence in Migration: An Introduction. Gender-Based Violence in Migration. Springer International Publishing; 2022. p. 3–28. doi: 10.1007/978-3-031-07929-0_1

[102] Alpysbekova A, Montero-Zamora P, Soares MH, Scaramutti C, Sahbaz S, Duque M, et al. A comparative study of Venezuelan immigrants’ pre- and post-migration concerns for their children in the United States and Colombia. PLoS One. 2024;19(12):e0313215. doi: 10.1371/journal.pone.0313215 39715235 PMC11665991

[103] Hankivsky O, Grace D, Hunting G, Giesbrecht M, Fridkin A, Rudrum S. An intersectionality-based policy analysis framework: critical reflections on a methodology for advancing equity. Int J Equity Health. 2014;13:119. 25492385 10.1186/s12939-014-0119-x PMC4271465

[104] Tan SE, Kuschminder K. Migrant experiences of sexual and gender based violence: a critical interpretative synthesis. Global Health. 2022;18(1):68. doi: 10.1186/s12992-022-00860-2 35765002 PMC9241205

