# Supplementary material for: Precarious employment and gender-based violence against migrant women: A scoping review mapping the intersections
Source: PLoS One. 2025 Dec 1;20(12):e0337690. doi: 10.1371/journal.pone.0337690 (PMC12668559; doi:10.1371/journal.pone.0337690)
Supplement: S1 Table — (DOCX) [file pone.0337690.s001.docx]

**APPENDICES**

**Appendix A: Medline Search Strategy**

| **Set** | **Search Statement** |
| --- | --- |
| 1 | exp Gender-Based Violence/ or (gender-based violence or gender-based violence or gender-based childhood violence or violence against women).tw,kf. |
| 2 | exp Intimate Partner Violence/ or (intimate partner violence or intimate partner abus* or partner abus* or partner violence or dating violence).tw,kf. |
| 3 | exp Domestic Violence/ or (domestic adj3 (abus* or violence)).tw,kf. |
| 4 | exp Spouse Abuse/ or ((spous* or wife or wive*) adj3 (abus* or violence*)).tw,kf. |
| 5 | exp Battered Women/ or (batter* adj3 (wife or wive* or spous* or wom?n)).tw,kf. |
| 6 | rape.tw,kf. Or exp Rape/ |
| 7 | exp Sex Offenses/ or (sex* adj3 (abus* or violence or crime or assault or harass*)).tw,kf. |
| 8 | exp Workplace Violence/ or ((workplace or occupation*) adj3 (abus* or violence or mistreatment)).tw,kf. |
| 9 | 1 or 2 or 3 or 4 or 5 or 6 or 7 or 8 |
| 10 | ((precarious or casual or informal or low wage or temporary or non standard or contract or on call or irregular or unstable or insecure or gig or agency or platform or season* or part time or care* or domestic or agricultu* or warehouse or construction or personal support) adj3 (employ* or job* or industry* or econo* or work* or labour or labor or help or vocation or career)).tw,kf. |
| 11 | exp Shift Work Schedule/ |
| 12 | exp Household Work/ |
| 13 | exp Caregivers/ |
| 14 | exp Manufacturing Industry/ or exp Food Industry/ or exp Industry/ or exp Textile Industry/ or exp Construction Industry/ or exp Meat-Packing Industry/ |
| 15 | 10 or 11 or 12 or 13 or 14 |
| 16 | “Emigration and Immigration”/ |
| 17 | exp “Emigrants and Immigrants”/ or (migrant* or immigrant* or expat* or foreign* or newcomer or tempor* resid* or permanent* resid*).tw,kf. |
| 18 | exp Refugees/ or asylum seek*.tw, kf. |
| 19 | 16 or 17 or 18 |
| 20 | 9 and 15 and 19 |

**Appendix B: Characteristics of included studies**

| **First author (Year) Country** | **Occupation** | **Participant group** | **Study design; Methods adopted** | **Immigration status** | **Study sample** | **Dimensions of occupational precarity^a^** | **GBV experience** |
| --- | --- | --- | --- | --- | --- | --- | --- |
| Anbesse (2009) Ethiopia | Domestic workers | MW | Qualitative inquiry: Interviews and focus group discussions | Temporary work visa | 19 returnee migrant women^e^ aged between 20 to 30 years | Long working hours, no protection, low wages, no workplace protections | Workplace physical, sexual and emotional abuse |
| Ayalon (2009) Israel | Home care workers | MW; SP | Survey questionnaire, qualitative inquiry-interviews, focus groups | NR^b^ | 29 women aged between 18 and 56 years.  31 social workers | Low wages/compensation, long working hours, no workplace protections | Workplace physical abuse; Emotional Abuse |
| Ayalon (2012) Israel | Home care workers | MW | Qualitative inquiry-focus group discussions; survey questionnaire | NR | 178 Filipino women with mean age of 37 years | Low wages/compensation, long working hours; no workplace protections | Workplace physical, sexual and emotional abuse  Other: food deprivation |
| Belanger (2014) Vietnam | Domestic workers; fisheries, agriculture, construction and cruise ship workers | MW | Qualitative inquiry: Interviews | NR | 646 returnee migrant women | Low wages/compensation, long working hours; no legal rights and benefits, no workplace protection | Workplace physical, emotional and sexual abuse; |
| Bevilacqua (2023) US | Informal jobs^d^ | MW | Qualitative inquiry: Interviews and focus group discussions | Permanent residents, temporary work visa, asylum status,  few chose not to disclose. | 22 women aged between 20 and 50 years. | Low wages/compensation, no workplace protections, no rights and benefits, long working hours | Workplace physical, sexual and emotional abuse |
| Bhuyan (2018) Canada | Caregivers | MW | Qualitative inquiry: Interviews and focus group discussions | Closed and open work permits, permanent residents. Undocumented | 31 women and 2 men aged between 26 and 58 years. | Long working hours, Low wages/compensation; no workplace protections; no rights | Workplace sexual and emotional abuse.  Other: food deprivation as a symbolic form of violence. |
| Boucher (2025)  Canada, Australia, United Kingdom, United States | Domestic workers, care workers/au pair, administrative assistants, farm workers | MW | Qualitative inquiry: 12 cases involving sexual violence | Temporary visa holders (e.g., international students, working holiday, temporary foreign worker; Undocumented | 12 women, age not specified | Low wages / unpaid work. No workplace protections, no rights | Workplace physical, emotional and sexual abuse; |
| Busza (2017) Ethiopia | Domestic workers | MW | Qualitative inquiry: Interviews and focus group discussions | NR | 35 women aged between 20 and 35 years. | Heavy workloads, no workplace protections, no rights and benefits | Workplace physical, emotional and sexual abuse |
| Cambara (2022)  Lao People’s Democratic Republic | Informal jobs | MW | Quantitative; Survey | NR | 405 Returnee migrant women aged between 18 and 51+ years. | Low wages/compensation; long working hours, no workplace protections, no benefits | Workplace Emotional abuse |
| Chan (2023) Chile | Domestic Workers | MW | Qualitative inquiry: Interviews | NR | 60 women; age not specified | long working hours; no workplace protections, no rights | Workplace physical and emotional abuse |
| Chan (2024)  Chile | Domestic workers | MW | Qualitative inquiry: interviews | NR | 3 women with age ranging between mid-30s and mid 40s | Long working hours, no workplace protections, no rights, low compensation/wages | Workplace physical, sexual and emotional abuse |
| Cheung (2019) China | Domestic Workers | MW | Quantitative: Survey | NR | 105 women aged 20 years and above. | No workplace protections; no benefits, no rights and heavy workloads | Workplace physical and emotional abuse |
| Chuemchit (2024)  Thailand | Service industry workers, factory workers, informal jobs | MW | Mixed methods: Survey; interviews and focus group discussions | Documented; undocumented | 572 women with median age 27 years | No workplace protections, low wages/compensation, excessive workloads | Workplace physical and emotional abuse, IPV |
| Chung (2020) China | Domestic workers | MW | Quantitative: Cross sectional | NR | 1945 Filipino and Indonesian women aged between 30-50 years | Low wages/compensation, long working hours, no workplace protections, no rights and benefits | Workplace physical and emotional abuse |
| Covington-Ward (2021) United States | Certified nursing  aides; home care aides, direct care/care providers, | MW | Qualitative inquiry: Interviews and focus group discussions | NR | 30 women aged between 22 and 53 years | Long working hours, no workplace protections, no rights | Workplace physical and emotional abuse |
| Diab (2022) Lebanon, Abhudhabi, Yemen | Domestic Workers | MW; SP | Qualitative inquiry: Interviews | Undocumented; documented | 913 women aged between 18 and 61 years. | Low or unpaid wages/overtime, heavy workloads, no workplace protections | Workplace sexual, physical and emotional abuse |
| Duke (2011) US | Farmworkers | MW | Quantitative: Survey | NR | 61 women, 37 men, 2 participants (gender not reported with an average age of 36 years. | Long working hours, heavy workloads, no workplace protections | IPV |
| Duque (2023) Spain | Domestic workers | MW | Qualitative inquiry: Interviews | Granted Citizenship; temporary work visa | 11 women aged between 36 and 59 years. | No workplace protections, long working hours, and heavy workloads, low wages/compensation | Workplace physical sexual and emotional abuse |
| Fuentes-Pumarola (2025)  Spain | Domestic workers; care workers | MW | Qualitative inquiry: interviews | Undocumented; documented | 28 women aged between 31 and 41 years | No workplace protections, low compensations/wages, long working hours, no contract, | Workplace physical, emotional and sexual violence |
| Gebreyesus (2018) Israel | Informal jobs | MW; SP | Qualitative inquiry: Interviews and focus group discussions | Asylum seekers | 56 migrant men and women and 25 key informants. Age range not provided | long working hours, no workplace protections, no right and benefits | Workplace physical, emotional and sexual abuse |
| Ghaddar (2020) Lebanon | Domestic Workers | SP | Qualitative inquiry: Interviews | NR | 42 agency managers between the ages of 35 and 50 years. | Heavy workloads, long work hours, no labour rights, no workplace protections | Workplace physical and emotional abuse |
| Gillespie (2022) Italy | Restaurant and, care workers, hotel maids or housekeepers | MW; SP | Qualitative inquiry: Interviews and focus group discussions | NR | 31 women aged between 18 and 65 years.  51 key informants. | No workplace protections, low to no wages/ compensation, no job security | IPV |
| Green (2016) Israel | Home care workers | MW | Quantitative: Survey | Temporary work visa | 85 women with an average age of 37 years. | No workplace protections, no rights low wages/compensation heavy workloads | Workplace physical, sexual and emotional abuse |
| Green (2017) Israel | Home care workers | MW | Quantitative: Survey | Temporary work visa | 187 women with an average age of 55 years. | No rights, no workplace protections, low wages, long working hours | Workplace, physical, sexual and emotional violence. |
| Green (2018) Israel | Home care workers | MW | Questionnaires.  Qualitative Inquiry: Face to face interview | NR | 338 women with an average age of 39 years | No workplace protections, no rights and benefits, long working hours, low wages/compensation | Workplace physical, sexual and emotional violence; |
| Hsieh (2017) US | Housekeepers | MW | Qualitative inquiry: Interviews | NR | 20 women aged between 22 to 52 years. | Low wages, long working hours, no paid overtime, unclear contracts, no workplace protections and no benefits, | Workplace emotional Abuse |
| Kim (2016) US | Farmworkers | MW | Qualitative inquiry: Focus group discussions | NR | 20 women aged between 19 and 68 years | Long working hours, no workplace protection, no rights and benefits, low wages | Workplace physical sexual and emotional abuse |
| Kodoth (2016) India | Domestic Workers | MW | Qualitative inquiry: Interviews Quantitative inquiry: Surveys | NR | 502 returnee migrant women, age range not provided | Excessive work loads, long working hours; no workplace protections, no rights and benefits | Workplace physical and emotional Abuse  Other: food deprivation |
| Kouta (2021) Cyprus | Domestic Workers, cleaners, care workers | MW | Qualitative inquiry: In depth individual and group interviews | NR | 15 women aged between 26 and 53 years | No workplace protections, no rights and benefits, low wages | Workplace sexual and emotional violence |
| Ladegaard (2025)  China | Domestic workers | MW | Qualitative inquiry: interviews ad focus group discussions | NR | 131 Women; Age range not provided | No workplace protections, low wages/compensation, long working hours | Workplace physical sexual and emotional abuse |
| Lai (2020) China | Domestic workers | MW | Quantitative: Survey | NR | 1750 women aged between 20 and 72 years | Long working hours, low wages, no workplace protection | Workplace physical and emotional violence |
| Mahdavi (2013) United Arab Emirates | Domestic Workers | MW | Qualitative inquiry: Ethnographic fieldwork | Undocumented^c^ | 72 women, age range not specified | Long working hours, low wages, no protections, no job security and benefits | Workplace physical and sexual abuse; Rape |
| Mondon-Navazo (2025)  Germany | Platform-based cleaning work | MW | Qualitative inquiry: Interviews;3 ,500 WhatsApp messages | Student visa, tourist visa, free lance visa, job seeking visa | 10 women aged between 29 and 35 years old | low compensation/wages, no formal workplace protections, long working hours | Workplace sexual and emotional abuse |
| Murphy (2015) US | Farmworkers | MW | Qualitative inquiry: Focus group discussions | NR | 49 women, age range not specified | No workplace protections, no rights and benefits | Sexual violence |
| Mutambara (2023) South Africa | Hairdressers, street vendors, informal tailors, seamstresses childminder. | MW | Qualitative inquiry: Interviews | Temporary work visa; undocumented | 22 migrant women aged between 25 and 49 years | Low wages/compensation, no workplace protections, no job security and benefits | IPV; Workplace physical and sexual abuse |
| Nahar (2021) US | Informal jobs | MW | Qualitative inquiry: focus group discussions | NR | 15 women aged between 25 and 68 years | Low wages/compensation, no workplace protections and no benefits, no job security | IPV |
| Ortega-de-Mora (2023) Spain | Cooks, dishwasher, warehouse worker, farm workers, care workers, restaurant workers | MW | Qualitative inquiry: Interviews | NR | 29 women; age range not specified | Job insecurity, no protection, long working hours, schedule unpredictability | Workplace physical, emotional and sexual abuse |
| Pan (2012) Taiwan | Domestic Workers | MW; SP | Qualitative inquiry: In-depth interviews | NR | 12 women between 20-40 years  10 staff members from NGOs/ government | Low wages/compensation, long working hours, no workplace protections, excessive workloads, schedule unpredictability | Workplace physical, sexual and emotional violence |
| Reda (2015) Ethiopia | Domestic workers; janitorial workers | MW; SP | Qualitative enquiry; focus group discussion, interviews | Undocumented | 67 Returnee migrant women, age range not specified. 25 key informants | Long working hours, schedule unpredictability, low wages, no workplace protection | Workplace physical violence, rape, emotional abuse |
| Robillard (2018) Canada | Not specified | SP | Qualitative inquiry: Interviews | NR | 47 service providers providing services to temporary foreign workers | Low wages/compensation, long working hours, no workplace protections, no rights | Workplace, sexual, physical and emotional abuse |
| Rocha (2022) US | Not specified | MW; SP | Qualitative inquiry: Interviews and focus group discussions | NR | 47 women aged between 22 and 74 years  16 key informants. | Long working hours, low wages/compensation, no protection, schedule unpredictability | IPV |
| Rodriguez-Martinez (2019) Spain | Domestic Workers | MW | Qualitative inquiry: Interviews | Undocumented | 32 women; age range not specified. | Long working hours, no workplace protections, schedule unpredictability, job insecurity | Workplace physical. emotional and sexual abuse |
| Serrano (2022)  Brazil | Garment workers | MW | Qualitative inquiry: in depth interviews, focus group discussions | NR | 17 women, age not specified | Long working hours, low wages/compensation, no workplace protections | IPV |
| Tran (2024) Saudi Arabia | Domestic workers | MW | Qualitative inquiry: interviews | Temporary work visa | 6 women aged between 16 and 40 years. | No workplace protections; long working hours, low compensation/wages, no rights | Workplace physical. emotional and sexual abuse |
| Tuncer (2022) Turkey | Domestic workers | MW | Qualitative inquiry: In-depth interviews | Undocumented; temporary work visa, | 12 women aged between 23 and 63 years | Long working hours, heavy workloads, schedule unpredictability, no protections, job insecurity | Workplace physical and sexual violence |
| Vasil (2023) Australia | Informal jobs | MW; SP | Qualitative inquiry: In-depth interviews | Dependent visa (partner/spousal, marriage visa); tourist visa, student visa; permanent resident | 18 women aged between 20 and 38 years.  21 key informants. | Long working hours, no rights and benefits, unsafe work conditions, schedule unpredictability | IPV |
| Villegas (2019) Canada | Restaurant workers; construction workers | MW | Qualitative inquiry: Interviews | Temporary work visa | 21 women, age range not specified | No workplace protections, no rights and, job insecurity | IPV; Workplace physical, sexual and emotional violence |
| Wickramage (2017) Sri Lanka | Domestic workers | MW | Qualitative inquiry: Interviews Quantitative inquiry: Survey | Temporary work visa | 20 women aged between 22 and 52 years. | Low to no wages/compensation, high workloads, long working hours, schedule unpredictability, unsafe working environments | Workplace physical, sexual and emotional abuse |
| Zahreddine (2014) Lebanon | Domestic workers | MW | Qualitative inquiry: Interviews Quantitative inquiry: Brief Psychotic Rating Scale (BPRS) and Clinical Global Impression (CGI) scales | Not reported | 33 women age between 20 and 50 years | Long working hours; Low wages/compensation, inhumane working and living conditions | Workplace physical violence |
| Zhang (2014) US | Agriculture, janitorial workers, food processing, and construction workers | MW | Quantitative: Survey | Undocumented | 826 migrant workers (405 identified as women with an average age of 33.5) | Long working hours, low wages/compensation, schedule unpredictability, no workplace protection | Workplace physical and emotional violence. |

**Abbreviations**: MW= migrant women; SP = service providers; NR= not reported; IPV=intimate partner violence; US= United States of America

^a^ This refers to the dimensions outlined in the studies that are used to describe occupational precarity

^b^ Migration status not reported by researchers or disclosed by participants

^c^ This refers to migrant women who did not have current legal authorization to be in the country- they may have entered the country illegally or overstayed their visas.

^d^ While these studies did not specify the actual jobs, they highlighted elements such as low wages that align with the dimensions of occupational precarity.

^e^ Women refers to migrant women, and when the focus is on those who have returned to their home countries, they are referred to as 'returnee migrant women.
